# A structured, indicator-driven quality improvement cycle is associated with improved adherence and outcomes after liver resection for hepatocellular carcinoma

**DOI:** 10.3389/fonc.2026.1785140

**Published:** 2026-04-10

**Authors:** Jiao Xie, Peng Guo, Yi Deng, Haiyun Shu, Xiu Du

**Affiliations:** Department of Hepatobiliary and Pancreatic Surgery, The Third Affiliated Hospital of Chongqing Medical University (Fangda Hospital), Chongqing, China

**Keywords:** audit and feedback, enhanced recovery, perioperative care, process metrics, surgical oncology

## Abstract

**Purpose:**

Despite established perioperative guidelines for hepatocellular carcinoma (HCC) resection, inconsistent implementation hinders optimal recovery. This study evaluated clinical outcomes associated with a structured, Nursing-Sensitive Indicator (NSI)-driven quality improvement program designed to ensure reliable execution of evidence-based practices.

**Patients and methods:**

We leveraged a cohort of 172 patients undergoing curative liver resection at The Third Affiliated Hospital of Chongqing Medical University from May 2019 to June 2024. An NSI-driven program featuring systematic monitoring, alert-triggered care bundles, and weekly audit/feedback was implemented. Program patients (Intervention, n=86) were compared to a historical usual-care cohort (Control, n=86) after 1:1 propensity score matching. Primary outcomes included perioperative process compliance and short-term recovery metrics (complications, length of stay). Secondary outcomes included 1-year recurrence-free survival (RFS) and patient-reported outcomes.

**Results:**

The intervention was significantly associated with improved process metric adherence, notably correlating with reduced time to first ambulation (17.8 ± 8.5 *vs*. 24.3 ± 9.9 hours, p<0.001) and increased pain assessment compliance (87.6 ± 5.1% *vs*. 77.3 ± 8.2%, p<0.001). This correlated with accelerated recovery, including shorter time to first flatus (59.2 ± 10.3 *vs*. 71.6 ± 13.8 hours, p<0.001) and postoperative stay (8.2 ± 1.8 *vs*. 10.3 ± 2.2 days, p<0.001). Severe complications (Clavien-Dindo ≥III) were numerically lower in the intervention group (10.5% *vs*. 18.6%, p=0.194), with notably lower overall infectious complications. Crucially, the intervention was significantly associated with improved 1-year (84.9% *vs*. 74.4%) and 2-year RFS (64.9% *vs*. 43.3%) (log-rank p=0.011). In multivariable analysis, NSI program enrollment remained independently associated with a reduced risk of recurrence (adjusted HR = 0.509, 95% CI: 0.314–0.824, p=0.006). Exploratory mediation analysis indicated 37.3% of the associated survival benefit might be mediated through reduced hospital stay.

**Conclusion:**

Implementing a structured NSI-driven quality management program was significantly associated with higher perioperative care fidelity, faster functional recovery, and better recurrence-free survival after HCC resection. This framework provides an effective mechanism for translating evidence-based guidelines into reliable routine practice, potentially correlating with favorable long-term oncological outcomes.

## 1 Introduction

Hepatocellular carcinoma (HCC) ranks among the most prevalent and life-threatening malignant neoplasms globally, with surgical resection remaining a cornerstone of curative-intent treatment for eligible patients with early-stage disease (1, 2). Despite advancements in surgical techniques and the establishment of evidence-based perioperative care guidelines, postoperative recovery following hepatectomy remains challenging (3). A notably high incidence of complications not only prolongs recovery but may also impair long-term survival outcomes. This discrepancy underscores a critical implementation gap between guideline recommendations and bedside practice, highlighting the need for effective systems to ensure consistent, high-fidelity care delivery.

To bridge this implementation gap, Nursing-Sensitive Indicators (NSIs) have emerged as pivotal tools for quantifying care quality and targeting improvement efforts (4, 5). Defined as measurable variables that reflect the quality and effectiveness of nursing care, NSIs enable the quantitative assessment and continuous improvement of clinical practice (4, 5). In HCC surgery, monitoring specific NSIs, which range from pain scores and time to mobilization to complication rates, provides a structured framework to evaluate care quality (6, 7). Studies demonstrate that interventions guided by such indicators can improve short-term outcomes, such as reducing hospitalization time and enhancing recovery in elderly HCC patients (1). Similarly, consensus guidelines from expert societies emphasize standardized perioperative management to reduce practice variation (3).

Recognizing the importance of standardized care, recent consensus recommendations, such as those from the E-AHPBA and ESSO, have been developed to provide evidence-based guidance on key aspects of perioperative management following liver resection. These guidelines address thromboprophylaxis, antibiotic use, prehabilitation, nutrition, mobilization, and the management of common complications like bile leaks and post-hepatectomy liver failure (3). The development of these guidelines highlights a concerted effort to standardize care and reduce practice variation. Similarly, the concept of “refined nursing strategies” in the ICU setting for post-laparoscopic HCC patients, which shares philosophical similarities with NSI-driven care, has been shown to accelerate postoperative recovery, improve liver function, and reduce complications (1). Furthermore, integrated rehabilitation models involving healthcare professionals, patients, and caregivers have also been reported to reduce adverse reactions and shorten recovery times (3, 8). While these guidelines delineate what constitutes optimal care, they often lack the operational framework necessary to ensure these components are consistently executed for every patient, thereby overcoming individual practice variation and clinical inertia.

However, a significant knowledge gap persists. Many initiatives utilizing NSIs focus on isolated care components or short-term rehabilitation metrics (6, 7). There is a comparative lack of robust evidence investigating whether a comprehensive, system-level application of NSIs, integrating real-time monitoring, triggered interventions, and audit feedback, can translate into improved long-term oncologic outcomes. The existing evidence often falls short of establishing a clear pathway from structured nursing care processes through intermediate NSIs to pivotal endpoints, such as tumor recurrence and survival.

Therefore, we aimed to investigate the clinical outcomes associated with a system-level, operational strategy utilizing a structured NSI-driven quality improvement program. This study tests the hypothesis that the implementation of such a data-feedback-action system correlates with standardized care processes, accelerated short-term recovery, and potentially favorable long-term oncologic outcomes for HCC patients undergoing resection. We specifically investigated its association with both short-term complications and, with particular emphasis, with long-term oncological outcomes, including one-year recurrence-free survival. We aim to contribute robust evidence to ongoing efforts aimed at standardizing and optimizing perioperative care pathways through measurable, system-level processes, ultimately correlating with comprehensive recovery and better long-term prognosis for patients undergoing curative surgery for hepatocellular carcinoma.

## 2 Materials and methods

### 2.1 Study population and eligibility criteria

A single-center, historical cohort study was conducted to evaluate the real-world effectiveness of a structured nursing quality management model driven by nursing-sensitive indicators (NSIs) for patients undergoing liver resection for hepatocellular carcinoma (HCC). This model was implemented as a departmental policy on June 1, 2021. Written informed consent was obtained from all participants. This research was performed in accordance with the Declaration of Helsinki and was approved by the Ethics Committee of The Third Affiliated Hospital of Chongqing Medical University (Fangda Hospital). The detailed Nursing-Sensitive Indicators and Standardized Response Protocol was summarized in **Supplementary Table 1**.

We retrospectively identified patients who underwent curative liver resection for HCC between May 2019 and June 2024. The inclusion criteria were: (1) age between 18 and 75 years; (2) histologically confirmed HCC; (3) underwent R0 radical hepatectomy. The exclusion criteria were: (1) presence of other synchronous malignancies; (2) evidence of extrahepatic metastasis before surgery; (3) severe preoperative liver dysfunction (Child-Pugh class C); (4) emergency surgery or combined multi-organ resection; (5) incomplete clinical records or loss to follow-up within 12 months postoperatively.

### 2.2 Cohort formation, follow-up, and propensity score matching

The cohort was divided based on the intervention date into a Control group (pre-intervention, treated from May 2019 to May 2021) and an Intervention group (post-intervention, treated from June 2021 to June 2024). To minimize selection bias and control for potential confounders, propensity scores were estimated for all patients using a logistic regression model that included key prognostic factors: age, gender, tumor number, maximum tumor size, presence of macrovascular invasion, BCLC stage (0/A *vs*. B), Child-Pugh grade (A/B), preoperative alpha-fetoprotein (AFP) level, microvascular invasion (MVI) status, surgical approach (laparoscopic *vs*. open), and receipt of any postoperative adjuvant therapy. Subsequently, each patient in the Intervention group was matched to one patient in the Control group using 1:1 nearest-neighbor propensity score matching with a caliper width of 0.2 standard deviations of the logit of the propensity score. Balance between the matched groups was assessed using standardized mean differences, with a threshold of <0.1 indicating good balance.

### 2.3 The NSI-driven quality improvement program: intervention and usual care

The exposure was cared under the formally implemented NSI-Driven Quality Improvement Program (NSI program). This program was designed as an operational system to enforce adherence to evidence-based perioperative care protocols. The core innovation was a closed-loop management system comprising: (1) systematic, shift-based monitoring using a standardized checklist of predefined NSIs; (2) a two-tiered electronic alert system (Yellow/Red) triggered by NSI thresholds, which mandated the execution of corresponding standardized care bundles; and (3) weekly multidisciplinary audit and feedback cycles to review compliance and outcomes.

To ensure seamless workflow integration, the standardized checklist and electronic alert system were directly embedded into the hospital’s existing electronic medical records (EMR) interface. Prior to implementation, nursing staff and clinicians completed a dedicated training module to familiarize themselves with the alert triggers and standardized response protocols. Existing staffing resources, including the routine ward nursing team and the primary surgical multidisciplinary team, successfully managed the program. Because it did not require the recruitment of dedicated quality-control personnel, this approach enhances potential reproducibility in other clinical settings.

The alert-triggered care bundles included: a) Pain Management Bundle (VAS assessment every 4 hours; scores ≥ 4); b) Early Recovery Bundle (protocol-driven first ambulation within 24 hours; structured interventions to stimulate gastrointestinal function); and c) Complication Prevention Bundle (standardized protocols for surgical site and drain care). In contrast, the Control group (usual care) involved similar clinical elements but delivered without the enforcing structure of the program. Practice relied on individual clinician discretion, lacking mandatory triggered responses and systematic audit, resulting in greater practice variability.

### 2.4 Data collection and outcome measures

Data were extracted from electronic medical records using a predefined, piloted data dictionary. Primary Outcomes: 1) 1-year recurrence-free survival (RFS), defined as the time from surgery to radiologically/histologically confirmed recurrence or death from any cause; 2) Incidence of postoperative complications within 90 days, graded using the Clavien-Dindo classification (Grade ≥ II considered significant). Secondary Outcomes: Included clinical recovery metrics (time to first ambulation, time to first flatus, postoperative length of stay) and patient-reported outcomes (pain intensity via VAS on POD 1, 3, and at discharge; quality of life via QOL-LC scale at admission and discharge). To assess the fidelity of the intervention, key process metrics were collected, including the proportion of patients achieving first ambulation within 24 hours and the proportion of documented pain alerts (VAS≥4) receiving intervention within 30 minutes.

### 2.5 Statistical analysis

After PSM, baseline characteristics were compared using t-test, Mann-Whitney U test, Chi-square, or Fisher’s exact test as appropriate. The primary time-to-event outcome, 1-year RFS, was analyzed using the Kaplan-Meier method with groups compared by the log-rank test. The independent association of the intervention with RFS was quantified using a multivariable Cox proportional hazards model applied to the matched cohort, adjusting for any residual imbalance in key covariates. Sensitivity analyses were pre-specified, including: 1) an analysis using the full, unmatched cohort with multivariable adjustment; 2) an analysis with a different PSM caliper (0.1); and 3) a complier analysis based on process indicator adherence in the intervention group. All analyses were conducted using R 4.2.2 and SPSS Statistics version 26.0, with a two-sided p-value < 0.05 considered statistically significant.

## 3 Results

### 3.1 Study population and baseline characteristics

**Figure 1:**
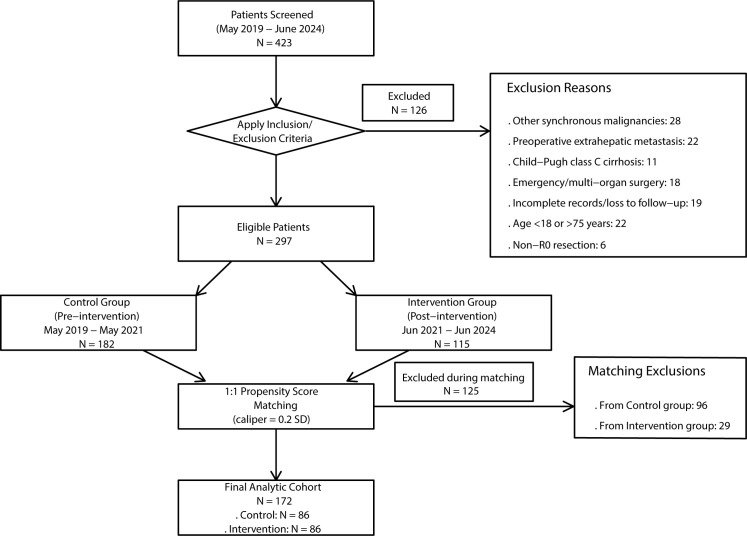
Flowchart of patient screening, eligibility, and cohort formation. This diagram illustrates the selection and matching process for the study evaluating a Nursing-Sensitive Indicator (NSI)-driven quality improvement program in patients undergoing liver resection for hepatocellular carcinoma (HCC).

A total of 172 patients with hepatocellular carcinoma (HCC) who underwent hepatectomy between May 2019 and June 2024 were included in the study, with 86 patients assigned to each of the Control (pre-intervention period: May 2019–May 2021) and Intervention (post-intervention period: June 2021–June 2024) groups. All patients were followed up until December 10, 2025 (**Figure 1**). As shown in **Table 1**, the matched cohorts were well-balanced with respect to key clinical and pathological prognostic factors, including age (Intervention: 57.1 ± 8.4 *vs*. Control: 57.4 ± 8.4 years, P = 0.849), sex distribution, tumor size and number, Child-Pugh class, BCLC stage, presence of microvascular invasion, and receipt of adjuvant therapy (all P > 0.05). A significant difference was observed in the surgical approach, with a higher proportion of open procedures in the Intervention group compared to the Control group (72.1% *vs*. 51.2%, P = 0.008); this variable was adjusted for in subsequent multivariable analyses.

**Table 1:** Baseline characteristics of HCC patients.

Variable	Level	Control	Intervention	P_Value
Age		57.4 ± 8.4	57.1 ± 8.4	0.849
Gender	Female	19 (22.1%)	22 (25.6%)	0.72
	Male	67 (77.9%)	64 (74.4%)	
Tumor_Size_cm		5.5 ± 2.4	5.0 ± 2.3	0.267
Tumor_Number	Single	63 (73.3%)	70 (81.4%)	0.275
	Multiple	23 (26.7%)	16 (18.6%)	
Child_Pugh_Class	A	74 (86.0%)	72 (83.7%)	0.831
	B	12 (14.0%)	14 (16.3%)	
BCLC_Stage	A	44 (60.3%)	47 (61.8%)	0.978
	B	29 (39.7%)	29 (38.2%)	
Microvascular_Invasion	Absent	60 (69.8%)	57 (66.3%)	0.744
	Present	26 (30.2%)	29 (33.7%)	
Surgical_Approach	Laparoscopic	42 (48.8%)	24 (27.9%)	0.008
	Open	44 (51.2%)	62 (72.1%)	
Adjuvant_Therapy	No	57 (66.3%)	58 (67.4%)	1
	Yes	29 (33.7%)	28 (32.6%)	

Data are presented as mean ± standard deviation for continuous variables and n (%) for categorical variables. The control group received usual care; the Intervention group was managed under the NSI-driven quality improvement program. P-values were derived from t-tests for continuous variables and chi-square tests for categorical variables. The only significant difference between groups after propensity score matching was in surgical approach (P = 0.008), which was adjusted for in subsequent multivariable analyses.

### 3.2 Adherence to process-of-care indicators (intervention fidelity)

**Figure 2:**
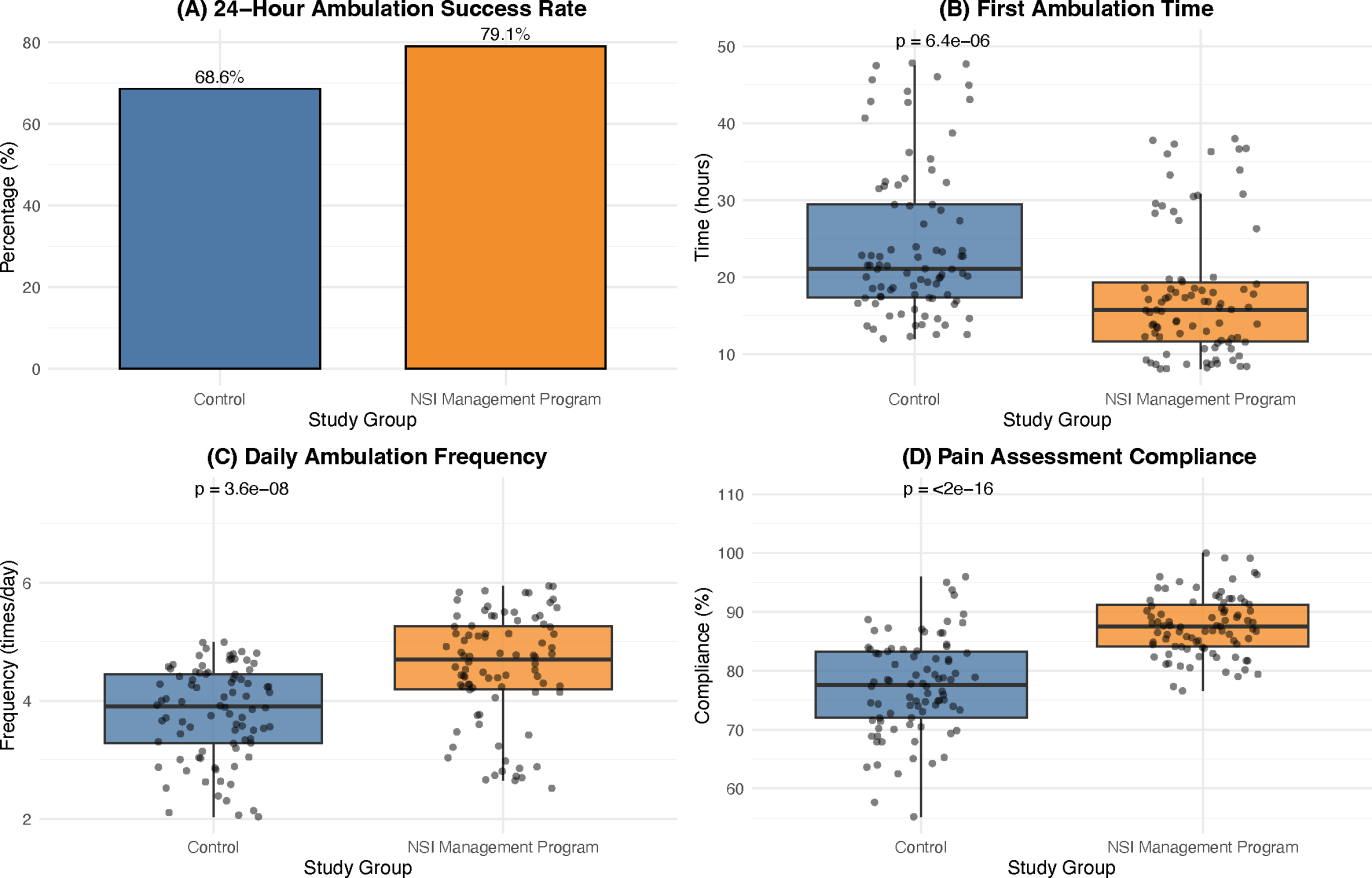
Process indicators analysis. Comparison of perioperative care process adherence between groups. **(A)** 24-hour ambulation success rate; **(B)** Time to first ambulation (hours); **(C)** Daily ambulation frequency; **(D)** Pain assessment compliance (%).

Implementation of the NSI program significantly improved adherence to key perioperative care processes (**Figure 2**). The median time to first ambulation was reduced by 6.5 hours in the Intervention group (17.8 ± 8.5 hours) compared to the Control group (24.3 ± 9.9 hours, P < 0.001). Consequently, the proportion of patients who ambulated within 24 hours increased to 79.1% in the Intervention group, compared to 68.6% in the Control group (P = 0.165). Patients in the Intervention group also performed daily ambulation more frequently (4.6 ± 0.9 *vs*. 3.8 ± 0.8 times/day, P < 0.001). Additionally, compliance with the standardized pain assessment protocol was significantly higher in the Intervention group (87.6 ± 5.1% *vs*. 77.3 ± 8.2%, P < 0.001).

### 3.3 Short-term clinical outcomes analysis

**Figure 3:**
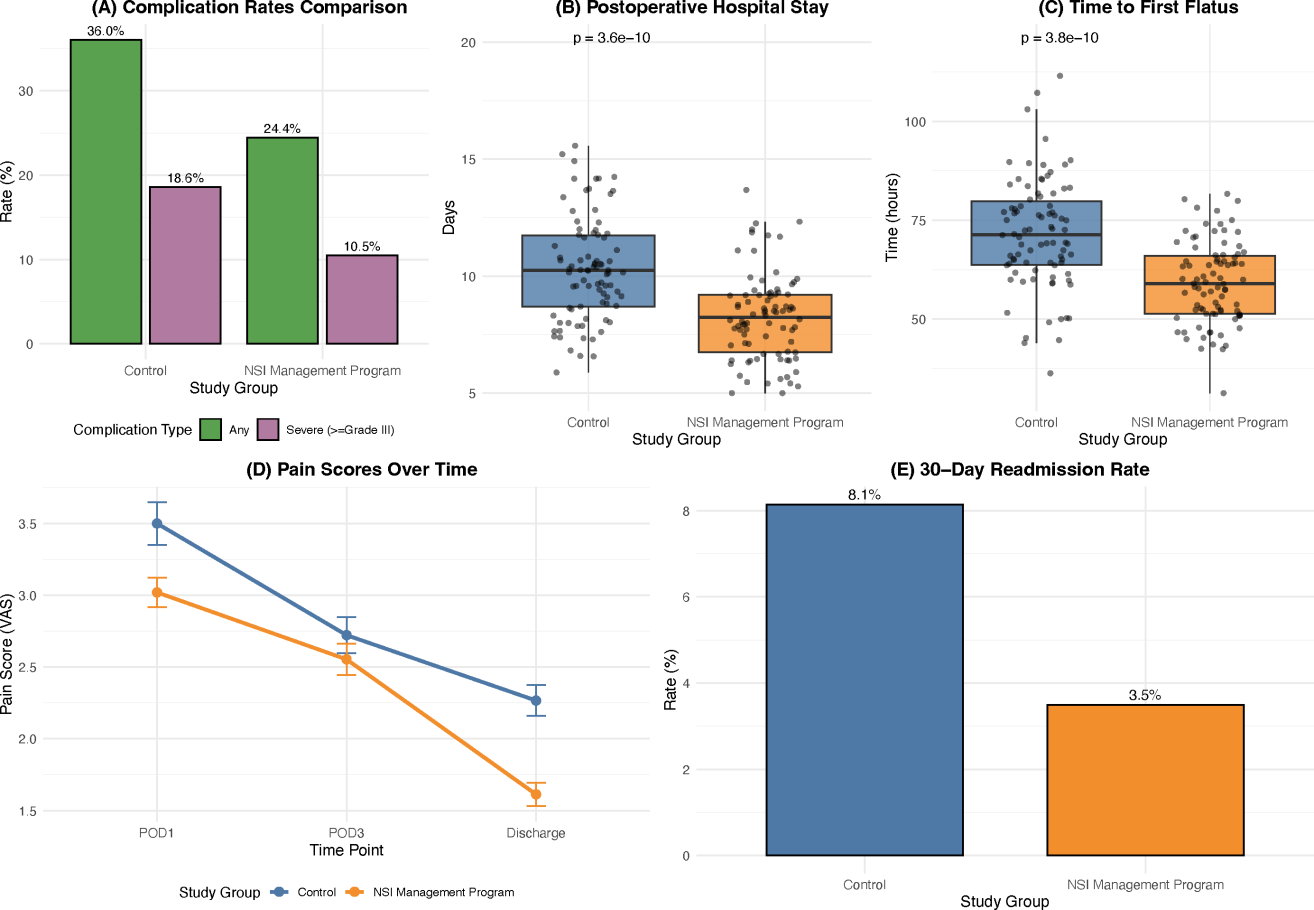
Short-term clinical outcomes analysis. Postoperative recovery and complication outcomes. **(A)** Complication rates by severity; **(B)** Postoperative hospital stay (days); **(C)** Time to first flatus (hours). **(D)** Pain scores over time (VAS); **(E)** 30-day readmission rates.

The improved process fidelity was associated with measurably accelerated postoperative recovery (**Figure 3**). The time to first flatus, a marker of gastrointestinal recovery, was significantly shorter in the Intervention group (59.2 ± 10.3 hours *vs*. 71.6 ± 13.8 hours, P < 0.001). The mean postoperative hospital stay was reduced by 2.1 days in the Intervention group (8.2 ± 1.8 days *vs*. 10.3 ± 2.2 days, P < 0.001). The incidence of severe complications (Clavien-Dindo ≥ Grade III) was lower in the Intervention group (9/86, 10.5%) than in the Control group (16/86, 18.6%), although this difference did not reach statistical significance (P = 0.194), potentially due to limited statistical power given the sample size. The 30-day readmission rate was also numerically lower (3.5% *vs*. 8.1%, P = 0.328). To better understand the clinical pathways affected, postoperative complications were further categorized by type (**Supplementary Table 2**). The reduction in overall complications in the Intervention group was primarily driven by a decrease in infectious complications, such as surgical site infections and pneumonia (9.3% *vs*. 16.3%), whereas the rates of liver-specific complications (e.g., bile leak, liver failure) remained similar between the cohorts.

### 3.4 Long-term oncological outcomes analysis

**Figure 4:**
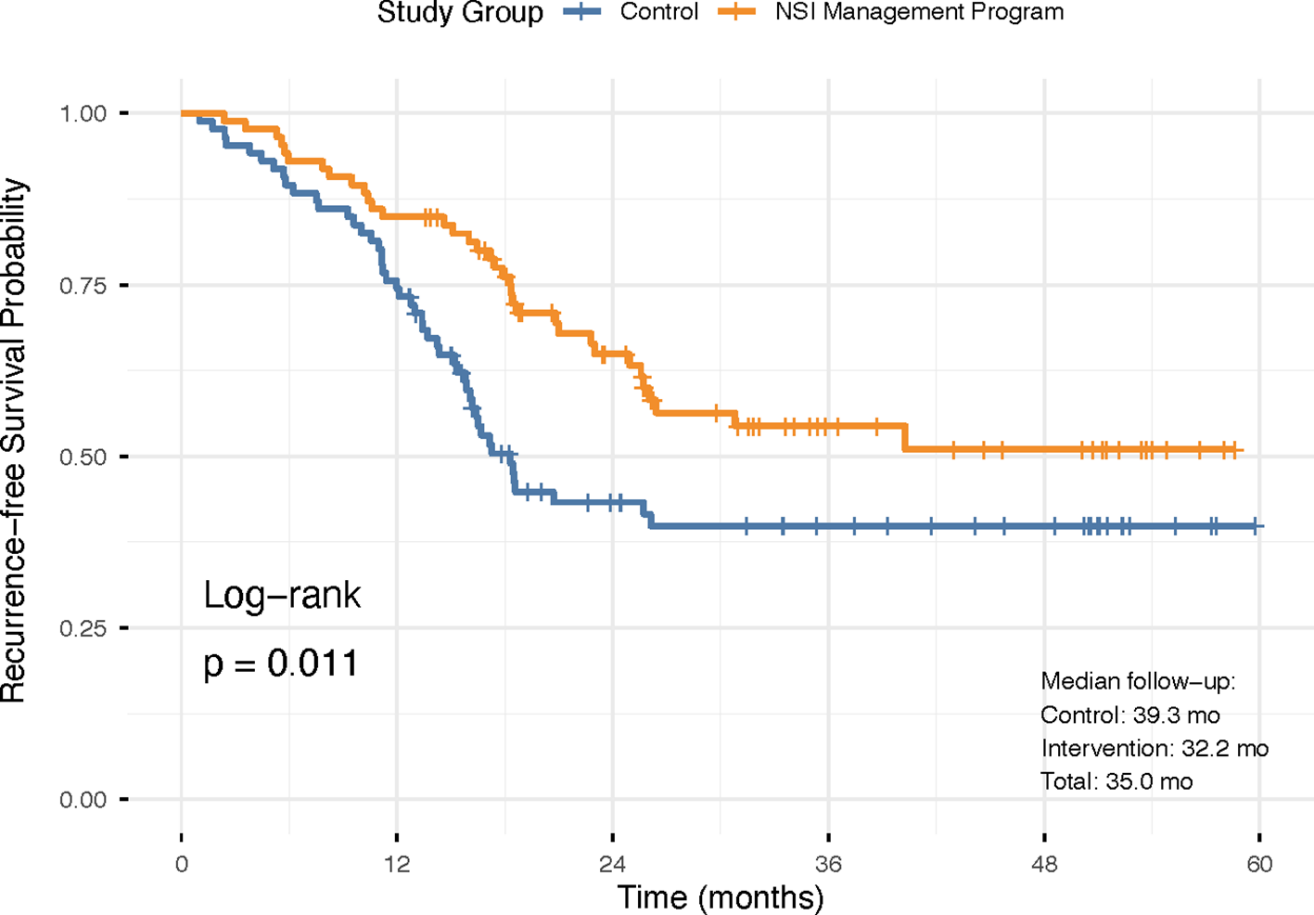
Recurrence-free survival analysis (Kaplan-Meier). Kaplan-Meier curves showing recurrence-free survival probability over time. The NSI program group (orange) demonstrated significantly better survival compared to the control group (blue) (log-rank P = 0.011).

With a median follow-up of 35.0 months overall (Intervention: 32.2 months, Control: 39.3 months), the NSI program was associated with superior recurrence-free survival (RFS) (**Figure 4**). The 1-year and 2-year RFS rates were 84.9% and 64.9% in the Intervention group, compared to 74.4% and 43.3% in the Control group, respectively. Kaplan-Meier analysis demonstrated a significant separation of the survival curves in favor of the Intervention group (log-rank P = 0.011). The unadjusted hazard ratio (HR) for recurrence or death was 0.572 (95% Confidence Interval [CI]: 0.369–0.886).

### 3.5 Multivariable Cox regression analysis

**Figure 5:**
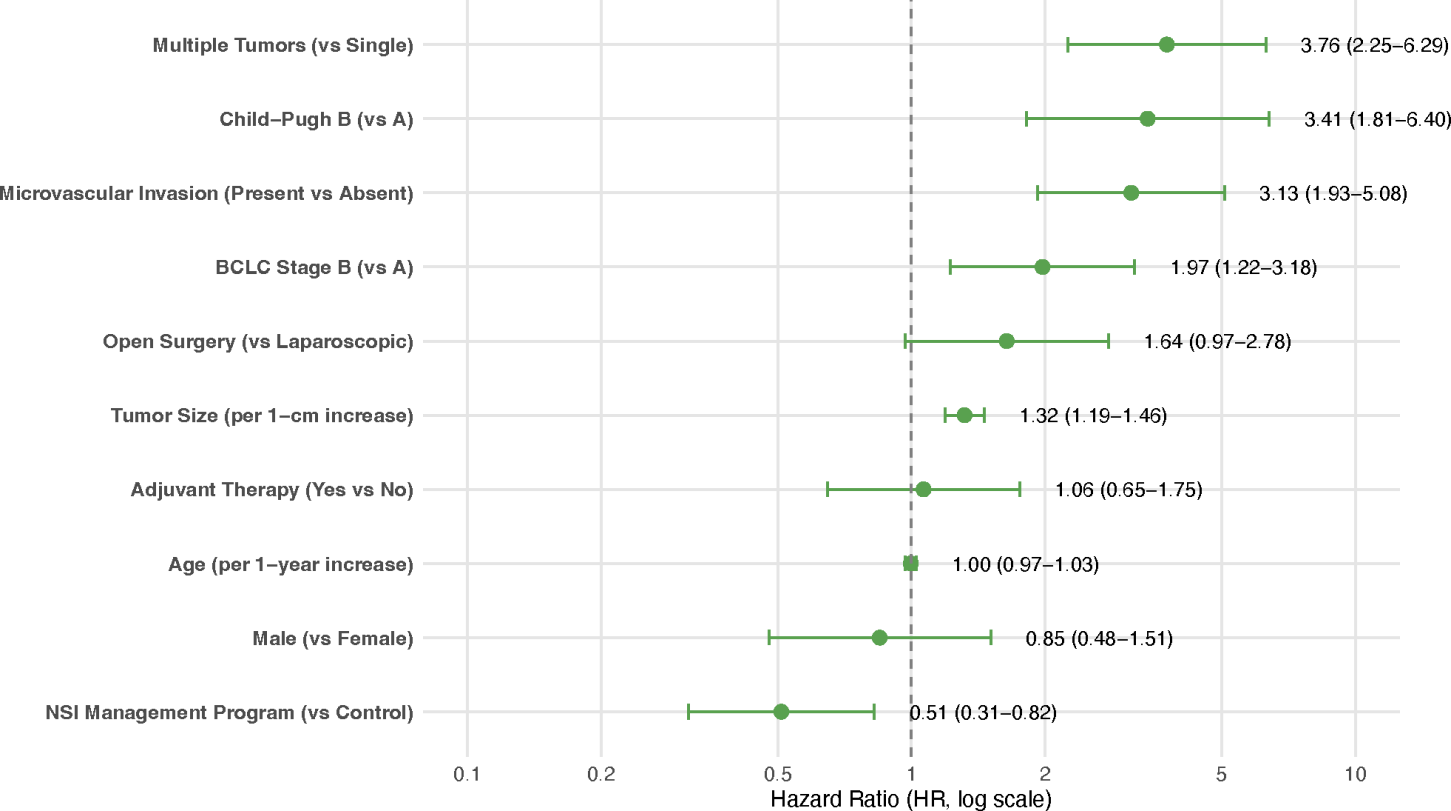
Multivariable Cox regression forest plot. Forest plot displaying adjusted hazard ratios (log scale) from the multivariable Cox regression model. Points represent point estimates; horizontal lines indicate 95% confidence intervals.

After adjusting for significant clinical and pathological covariates in a multivariable Cox proportional hazards model, enrollment in the NSI management program remained independently associated with a reduced risk of recurrence or death (Adjusted HR = 0.509, 95% CI: 0.314–0.824, P = 0.006) (**Table 2**, **Figure 5**). Other independent factors associated with a significantly increased risk of recurrence included larger tumor size (per 1-cm increase: HR = 1.317, 95% CI: 1.189–1.459, P<0.001), multiple tumors (*vs*. single: HR = 3.763, 95% CI: 2.250–6.294, P<0.001), Child-Pugh B cirrhosis (*vs*. A: HR = 3.406, 95% CI: 1.813–6.396, P<0.001), BCLC stage B (*vs*. A: HR = 1.974, 95% CI: 1.224–3.184, P = 0.005), and the presence of microvascular invasion (HR = 3.128, 95% CI: 1.927–5.078, P<0.001).

**Table 2:** Multivariable Cox regression analysis results.

Variable	HR	CI_95	P_Value
NSI Program (*vs* Control)	0.509	0.314-0.824	0.006
Age (per 1-year increase)	0.997	0.969-1.026	0.8624
Male (*vs* Female)	0.848	0.477-1.508	0.574
Tumor Size (per 1-cm increase)	1.317	1.189-1.459	<0.001
Multiple Tumors (*vs* Single)	3.763	2.250-6.294	<0.001
Child-Pugh B (*vs* A)	3.406	1.813-6.396	<0.001
BCLC Stage B (*vs* A)	1.974	1.224-3.184	0.0053
Microvascular Invasion (Present *vs* Absent)	3.128	1.927-5.078	<0.001
Open Surgery (*vs* Laparoscopic)	1.639	0.967-2.779	0.0665
Adjuvant Therapy (Yes *vs* No)	1.065	0.648-1.752	0.803

Adjusted hazard ratios for recurrence-free survival from the Cox proportional hazards model. The NSI program remained independently associated with reduced recurrence risk (HR = 0.509, 95% CI: 0.314-0.824, P = 0.006) after adjustment for key clinical and pathological factors. Other significant predictors included tumor size, tumor number, Child-Pugh class, BCLC stage, and microvascular invasion.

### 3.6 Mediation analysis

**Figure 6:**
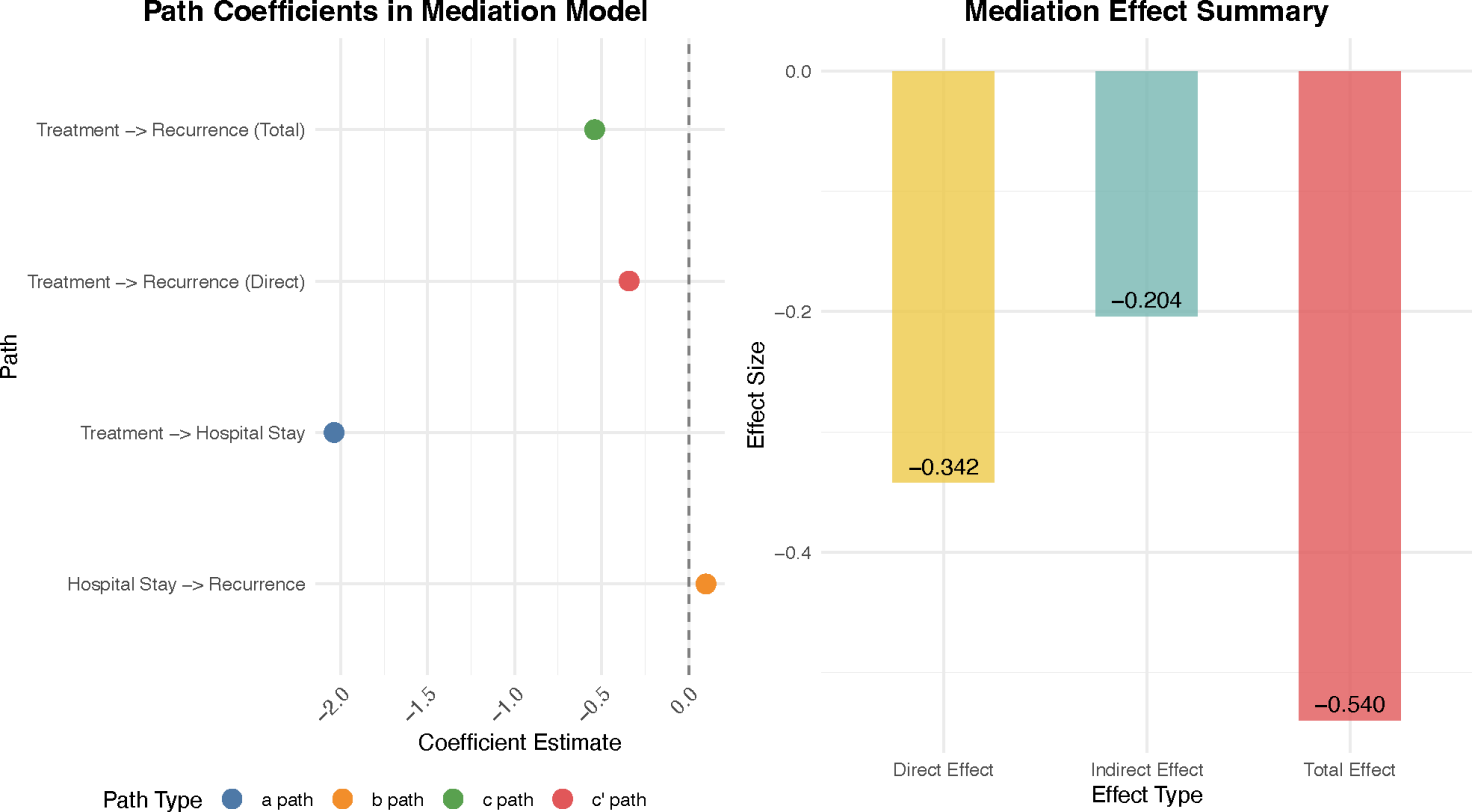
Mediation analysis: hospital stay as mediator. Path analysis examining the mediating role of postoperative hospital stay. Left panel: Path coefficient estimates for each relationship in the mediation model. Right panel: Decomposition of total associated benefit into direct and indirect components.

To explore potential hypothesized pathways associated with the intervention, an exploratory mediation analysis was conducted (**Figure 6**). The analysis revealed that a significant proportion of the associated benefit of the NSI program on reducing recurrence risk was mediated through the shortening of postoperative hospital stay. The indirect (mediated) effect was estimated at -0.204, accounting for 37.3% of the total associated benefit (Total Effect: -0.540). The direct effect associated with the intervention remained significant (-0.342), indicating the program was associated with a benefit beyond merely reducing hospital stay. However, because the length of stay is a multifactorial variable that may also reflect postoperative complications and patient frailty rather than functioning solely as a direct causal mediator, these mediation findings should be interpreted as hypothesis-generating.

### 3.7 Subgroup analysis results

**Figure 7:**
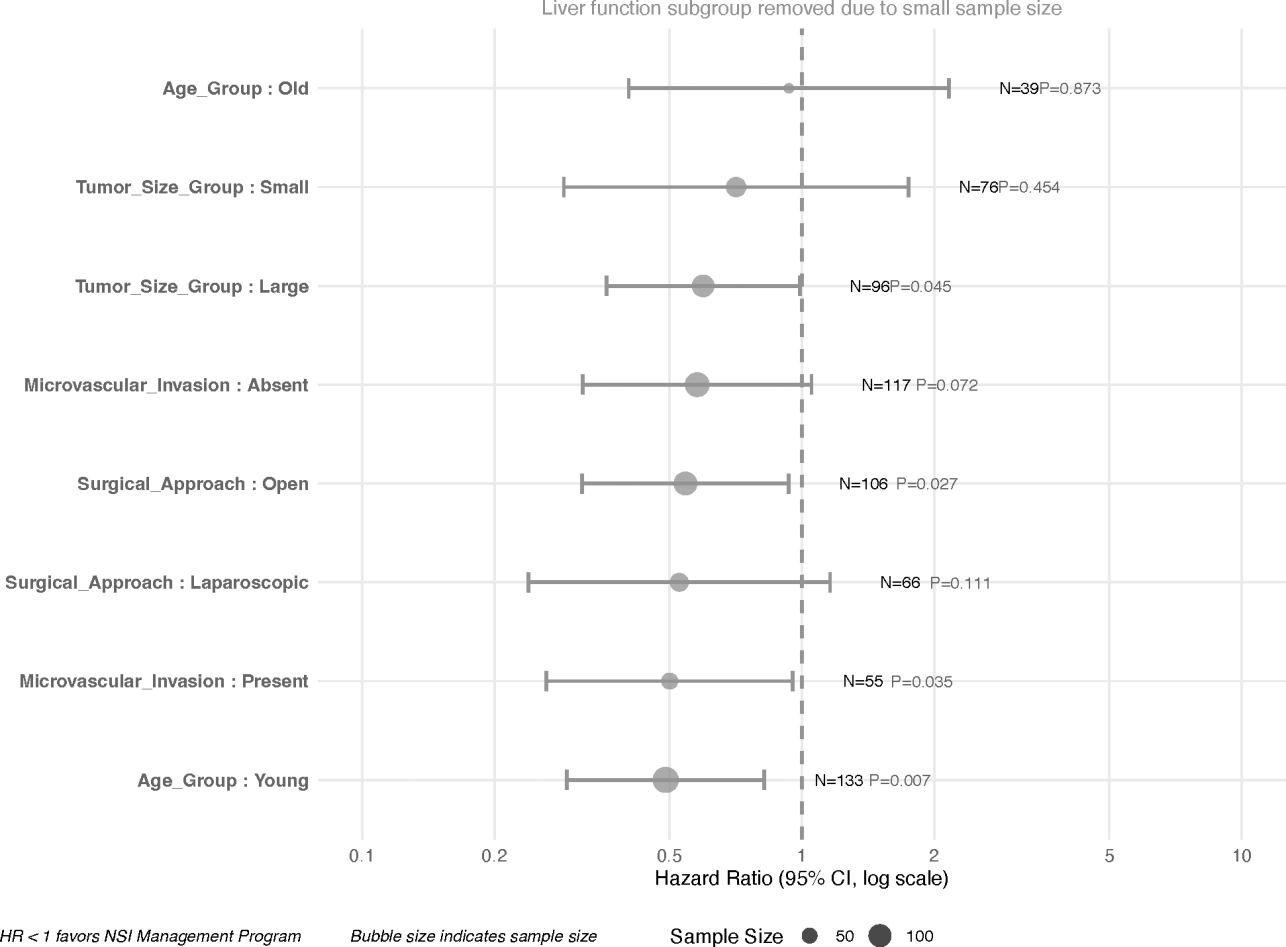
Subgroup analysis forest plot. Forest plot showing hazard ratios for recurrence-free survival across patient subgroups, with points colored by subgroup category and sized by sample size. Horizontal lines represent 95% confidence intervals.

Consistency of the associated benefit was assessed across key clinical subgroups (**Table 3**, **Figure 7**). The benefit of the NSI program (HR < 1) was observed consistently across most subgroups. The reduction in recurrence risk was statistically significant in several high-risk subgroups, including younger patients (HR = 0.49, 95% CI: 0.29–0.82, P = 0.007), patients with microvascular invasion (HR = 0.50, 95% CI: 0.26–0.95, P = 0.035), those undergoing open surgery (HR = 0.54, 95% CI: 0.32–0.93, P = 0.027), and patients with larger tumors (HR = 0.60, 95% CI: 0.36–0.99, P = 0.045). The point estimate also favored the intervention in the laparoscopic surgery subgroup, though it was not statistically significant (HR = 0.53, 95% CI: 0.24–1.16, P = 0.111). No significant benefit was observed in subgroups of older patients or those with small tumors. Furthermore, it should be noted that some subgroups had smaller sample sizes, which resulted in wide confidence intervals; thus, these specific subgroup findings should be interpreted with appropriate caution.

**Table 3:** Subgroup analysis results.

Subgroup	N_Total	HR_95CI	P_Value_formatted
Age_Group: Young	133	0.49 (0.29-0.82)	0.007
Microvascular_Invasion: Present	55	0.50 (0.26-0.95)	0.035
Surgical_Approach: Laparoscopic	66	0.53 (0.24-1.16)	0.111
Surgical_Approach: Open	106	0.54 (0.32-0.93)	0.027
Microvascular_Invasion: Absent	117	0.58 (0.32-1.05)	0.072
Tumor_Size_Group: Large	96	0.60 (0.36-0.99)	0.045
Tumor_Size_Group: Small	76	0.71 (0.29-1.75)	0.454
Age_Group: Old	39	0.93 (0.40-2.16)	0.873

Hazard ratios for recurrence-free survival in predefined patient subgroups. The associated benefit of the NSI program was particularly pronounced in high-risk subgroups, including younger patients, those with microvascular invasion, open surgery patients, and those with larger tumors. The liver function subgroup was excluded due to a small sample size (n=26).

## 4 Discussion

This study evaluated the clinical outcomes associated with a structured, Nursing-Sensitive Indicator (NSI)-driven quality improvement program following liver resection for HCC. The primary findings indicate that the systematic implementation of this program was significantly associated with higher adherence to key perioperative process metrics. This greater adherence correlated with faster functional recovery, shorter postoperative length of stay (LOS), and better 1- and 2-year RFS. An exploratory mediation analysis further revealed that shortened hospital stay might serve as a mediating pathway. These results provide evidence supporting a data-feedback-action framework that effectively bridges the gap between established clinical guidelines and routine bedside practice (9), offering a practical model for optimizing care delivery, which may correlate with better recovery trajectories and oncological outcomes in surgical oncology.

A key contribution of this study lies in highlighting the potential link between standardized nursing care processes, short-term clinical recovery, and long-term oncological benefits. At the process level, the closed-loop NSI program effectively reduced clinical inertia and minimized practice variation. By translating evidence-based measures into actionable electronic alerts and standardized care bundles, we achieved a high degree of homogenization in clinical care delivery. This approach is consistent with the increasing adoption of standardized nursing frameworks (e.g., NNN linkages) designed to unify nursing practices and guide targeted interventions (10). Crucially, this enhanced process fidelity was associated with tangible short-term patient benefits, such as earlier ambulation and quicker return of gastrointestinal function, which are core tenets of Enhanced Recovery After Surgery (ERAS) protocols known to correlate with favorable outcomes in hepatobiliary surgery (11).

The most notable finding is the association between the NSI program and superior recurrence-free survival (RFS). Following rigorous adjustment for known prognostic factors using propensity score methods (12), program enrollment remained an independent protective factor. Importantly, while propensity matching was utilized, a significant imbalance remained regarding the surgical approach, with a higher proportion of open surgeries in the intervention group. Because open hepatectomy is traditionally associated with greater surgical trauma and a prolonged recovery compared to laparoscopic approaches, this baseline imbalance would theoretically bias the results against the intervention. The observation that patients in the NSI program still achieved faster recovery and better RFS despite higher rates of open surgery suggests a robust positive association with the intervention, acting as a bias toward the null. These results position high-fidelity perioperative management as a potentially modifiable prognostic factor, a notion corroborated by advances in surgical technique, such as the strong association between achieving a “textbook outcome” and better long-term survival (13, 14).

The exploratory mediation analysis provides a hypothesized mechanistic explanation for how a nursing-oriented intervention might be linked to tumor recurrence. The observation that reduced postoperative LOS mediated 37.3% of the associated survival benefit is a notable finding. However, LOS is a multifactorial variable that reflects not only the speed of functional recovery but also patient frailty and the occurrence of specific postoperative complications. Indeed, predictive models confirm that a patient’s physiological reserve and surgical factors are key drivers of LOS, directly connecting the biology of recovery to time in hospital (15). Prolonged hospitalization often signals suboptimal recovery and a persistent pro-inflammatory, immunosuppressive state (13). Our detailed complication analysis supports this; the reduction in overall complications was primarily driven by a decrease in infectious complications (e.g., pneumonia and surgical site infections), while liver-specific complications remained comparable between groups. This view is supported by wider surgical oncology evidence, which shows that postoperative morbidity and its attendant physiological stress can adversely affect long-term survival (16). The NSI program likely mitigates these risks by promoting early mobilization and effective pain control, thereby potentially attenuating the postoperative stress response and systemic inflammation. It is hypothesized that this moderation of physiological disturbance may render the microenvironment less favorable for the growth of residual micrometastatic cells. Nevertheless, because our study did not directly measure biological intermediates such as inflammatory cytokines or immune cell profiles, this mechanistic interpretation remains speculative and warrants future biological validation.

Our results align with two key contemporary shifts in oncology: the systematization of care delivery and the personalization of treatment. For instance, just as modern multidisciplinary teams (MDTs) employ prognostic tools like the CABLE score to guide first-line immunotherapy in advanced HCC (17), our NSI program utilizes standardized frameworks at the bedside to optimize surgical recovery. Furthermore, our focus on measurable processes resonates with global initiatives to define core quality indicators (QIs) for HCC (18). Furthermore, system-level factors must be considered; broader hospital characteristics, such as procedural volume and teaching status, are known to influence postoperative outcomes (19). Preventative strategies for recurrence remain a multifaceted endeavor, encompassing optimal initial therapy selection (20), neoadjuvant approaches (21), and dynamic, biomarker-driven surveillance models (22, 23). Future NSI program could potentially incorporate alerts triggered by such biomarker thresholds.

Several limitations should be noted. First, despite statistical adjustments, the historical cohort design inherently limits definitive causal inference, as it cannot eliminate unmeasured confounding or secular trends in surgical technique and perioperative management over time. Second, while the sample size was sufficient for the primary outcome, it was relatively limited for secondary endpoints; specifically, the study may have been underpowered to detect statistically significant differences in severe major complications (Clavien-Dindo ≥ III). Additionally, small sample sizes in certain subgroups led to wide confidence intervals, requiring these specific findings to be interpreted with caution. Third, the specific effect of the care “bundle” is difficult to isolate. Fourth, as this study utilized a locally developed NSI program, its external validity and reproducibility in institutions with different staffing models or electronic medical record capabilities require further validation. Future research should focus on several key directions to build upon this work. First, prospective multicenter validation is needed (19), alongside the integration of biomarkers with physiological recovery metrics to illuminate biological pathways (22). Second, it is important to examine whether optimized perioperative care enhances the efficacy or tolerability of emerging adjuvant and combination therapies, including modern targeted and immunotherapy regimens (24). Finally, evaluating the feasibility, safety, and cost-effectiveness of advanced care models, such as ERAS-based ambulatory liver surgery, would test the logical extreme of hospitalization reduction (25, 26). Dedicated cost-effectiveness and implementation science analyses are essential to identify systemic barriers to the widespread adoption of such data-driven quality systems.

## 5 Conclusion

In conclusion, the implementation of a structured NSI-driven quality improvement program was associated with higher fidelity of perioperative care for patients undergoing liver resection for HCC. This correlated with faster recovery, shorter hospital stays, and a clinically meaningful advantage in recurrence-free survival. Exploratory analyses suggest that a substantial portion of this survival benefit may be mediated through the achievement of a shorter, more uncomplicated postoperative course. This study provides a practical, operational framework for translating clinical guidelines into reliable routine practice, demonstrating that systematic, measurement-based nursing care has the potential to correlate with benefits extending beyond immediate recovery to potentially align with favorable long-term cancer outcomes.

## Data Availability

The original contributions presented in the study are included in the article/**Supplementary Material**. Further inquiries can be directed to the corresponding author.
